# Potential S1 Nerve Root Blocks Associated with Sacroiliac Joint Injections

**DOI:** 10.1155/2024/8064804

**Published:** 2024-07-30

**Authors:** Andrew Ng, Jesse Lou, Dajie Wang

**Affiliations:** Department of Anesthesiology Jefferson Pain Center Sidney Kimmel Medical College at Thomas Jefferson University, Philadelphia, Pennsylvania, USA

## Abstract

**Background:**

Sacroiliac (SI) joint dysfunction is a common cause of lower back pain. The diagnosis of SI joint pain remains challenging. Sacroiliac joint injection remains the gold standard of diagnosis of SI joint pain as well as providing therapeutic effect. One complication related to SI joint injection is temporary numbness and weakness of the leg.

**Objectives:**

To evaluate the anatomy of the SI joint and the flow of the contrast in the sacroiliac joint and to understand how local anesthetic can affect the nerve roots and cause temporary weakness and numbness of the leg. *Study Design*. Retrospective case series. *Setting*. Academic medical center.

**Methods:**

Patients who underwent SI joint injection with three-dimensional cone beam computed tomography with fluoroscopy (3D-CBCT) imaging were identified through retrospective review of two providers' case log from the electronic medical record. The cone beam CT images were reviewed to study the contrast spread and flow in the SI joint.

**Results:**

27/32 patients with the mean age of 56 years (range 39–87 years), 20 females, and 7 males were included in this study. After reviewing cone beam CT images, 4/27 (14.8%) patients showed contrast spread in the SI joint and spread into the S1 posterior neuroforamen. The remainder 23/27 (85.2%) patients had contrast localized in the SI joint. *Limitations*. Small population size, retrospective review of medical records.

**Conclusion:**

Our results indicate that the injection of lower concentration of local anesthetic with less volume may be necessary to decrease the risk of S1 nerve root block and epidural block. Furthermore, to improve the specificity of a diagnostic SI injection, an appropriate evaluation should be considered to rule out any S1 nerve pathology as a significant pain generator.

## 1. Introduction

Sacroiliac (SI) joint pain is a common cause of lower back pain with approximately 15 to 30 percent of lower back pain originating from the SI joint [1, 2]. SI joint pain is typically localized in the lower back and buttocks. However, pain in the SI joint region has a wide range of differential diagnosis, which includes pain from lumbar spine, muscles, ligaments, and hip joints, as well as visceral pain [3]. An inaccurate or incorrect diagnosis will undoubtedly lead to treatment failure. The diagnosis of SI joint pain remains challenging in our daily practice. Common physical examinations for SI joint pain include Fortin finger, sacral distraction, sacral compression, thigh thrust, FABER/Patrick, Gaenslen's, and tenderness over the posterior superior iliac spine (PSIS) [4, 5]. These physical examinations were designed to reproduce SI joint pain with provocation and palpation maneuvers. However, these tests have high rates of false positives and negatives with variable rates of sensitivity and specificity [5, 6]. History and physical examinations in conjunction with imaging studies can be helpful in the diagnosis of SI joint pain [7]. Currently, the gold standard for diagnosis of SI joint dysfunction is an intra-articular injection of a local anesthetic with or without corticosteroids [2, 8].

SI joint is unique due to its multiplanar orientation, irregular joint gap, partial ankylosis, and thick dorsal and interosseous ligaments. The joint space can be difficult to assess without image guidance. Sacroiliac joint injection can be performed utilizing fluoroscopy, ultrasound, and computed tomography (CT) [9, 10]. The commonly adopted guidance technology in clinical practice is fluoroscopy. However, because of multiplanar orientation of the joint and irregular joint gap, it can be difficult to access the joint space with traditional fluoroscopic guidance alone [11]. CT scan, with its cross-sectional images, can identify posterior joint gap, which allows interventionalist to find the optimal target to access the joint space. Several studies concluded that CT guidance is the best method of precise needle placement into the SI joints [12–14]. In this retrospective case review, all SI joint injections were performed under three-dimensional cone beam computed tomography with fluoroscopy.

Although SI joint injection is a relatively safe procedure, there are associated complications of exacerbation of pain, vasovagal reactions, injection site soreness, and facial flushing and/or sweating. Another complication is temporary numbness and weakness in the leg [15]. This weakness is likely associated with nerve block and should resolve within a few hours after the procedure depending on the type of local anesthetic used. However, the exact mechanism of leg weakness associated with SI joint injection is poorly understood.

Current conservative treatment modalities for SI joint pain include medications, bracing, physical therapy, SI joint injections and radiofrequency ablation of the L5 dorsal ramus, and lateral branches of the S1, S2, and S3 [4, 15]. If these conservative modalities fail, the next step is sacroiliac joint fusion. There is mounting evidence that minimally invasive sacroiliac joint fusions have provided clinically significant improvements in pain scores and disability [16, 17]. However, the success of the SI joint fusion is dependent on the accuracy and specificity of SI joint injection. This retrospective review aims to evaluate the contrast spread from SI joint injection with three-dimensional cone beam computed tomography.

## 2. Methods

This study was determined to be exempt from review from The Office of Human Research Institutional Review Board of Thomas Jefferson University. A retrospective review of the procedure database of two pain fellowship trained pain physicians was conducted to identify all patients that underwent sacroiliac joint injections with three-dimensional cone beam computed tomography with fluoroscopy (3D-CBCT) imaging (March 2021 – September 2022) utilizing electronic medical record. The following exclusion criteria were used–patients with prior history of sacroiliac joint fusion or sacral fracture, patients who underwent repeat injection with the same imaging guidance, patients who did not receive contrast into the joint space, and patients with a prior history of L5/S1 level surgery. The patients who received bilateral injections were considered as two separate sacroiliac joint injections. The acquired cone beam CT images of included patients were reviewed to study the contrast spread in the sacroiliac joints and evaluate the flow of contrast. Three observers reviewed the images independently (D.W., A.N, and J.L.). Disagreement was resolved by consensus.

### 2.1. Technique

The patients were evaluated and examined in the office for lower back pain. Sacroiliac joint dysfunction was diagnosed based on clinical symptoms and physical examinations with at least three provocative maneuvers. The patients were recommended sacroiliac joint injections. All patients had appropriate informed consent, and they underwent SI joint injections under 3D-CBCT (GE Discovery iGS7 with 3D image fusion capability). For the procedure, the patient was positioned prone. A CT scan was performed. The posterior joint space was visualized on axial view. Our target is typically at the junction of superior one-third and inferior two-thirds of the joint, which is an area with a relatively large posterior joint opening and in the synovial part of joint space. The CT navigation system was used to target the joint space. The needle entry site was anesthetized with lidocaine 1%. A 22 G spinal needle was used to advance into the sacroiliac joint under fluoroscopic guidance. One mL of contrast was injected through the needle. Cone beam CT images were acquired again to evaluate accuracy of needle placement and contrast spread in the SI joint. Once the needle placement is confirmed, 4 mL of mixture of 3 ml bupivacaine 0.25% and 1 ml (6 mg of betamethasone injectable suspension) was injected.

## 3. Results

**Figure 1:**
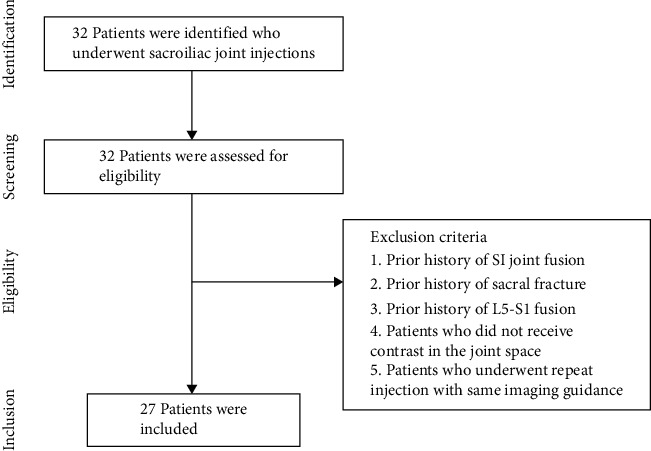
Exclusion criteria for this study.

**Figure 2:**
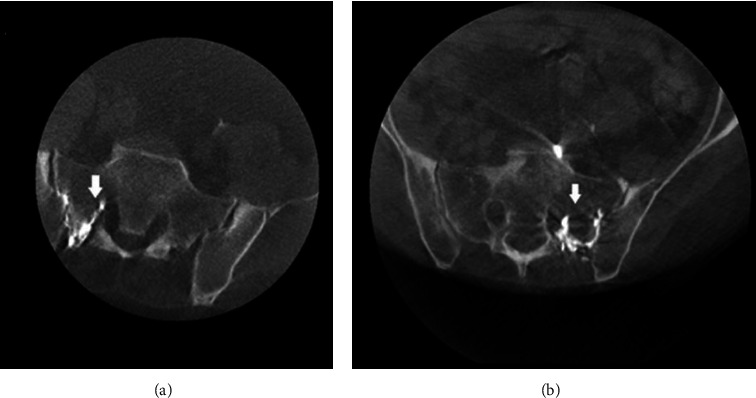
(a, b) 3D-CBCT images of two patients who received sacroiliac joint injections (the arrow indicates contrast spread from sacroiliac joint into posterior S1 neuroforamen).

**Figure 3:**
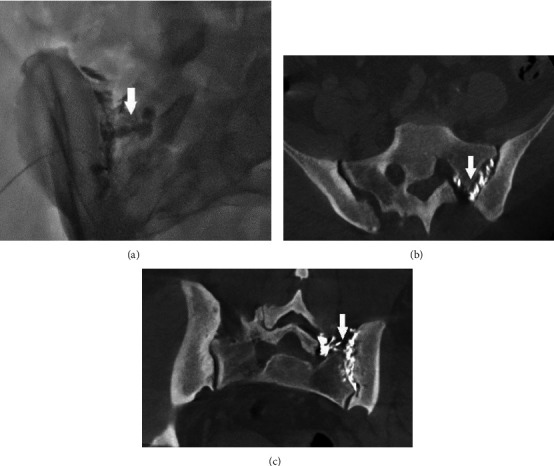
(a): Contrast spread from sacroiliac joint to S1 neuroforamen on an oblique view on the fluoroscopic image. (b): 3D-CBCT image, axial view of the sacroiliac iliac joint. Contrast spread in the sacroiliac joint and S1 neuroforamen. (c): 3D-CBCT image, coronal view of sacroiliac joint. Contrast spread in the sacroiliac joint and S1 neuroforamen (arrow indicating contrast spread into the S1 neuroforamen).

**Figure 4:**
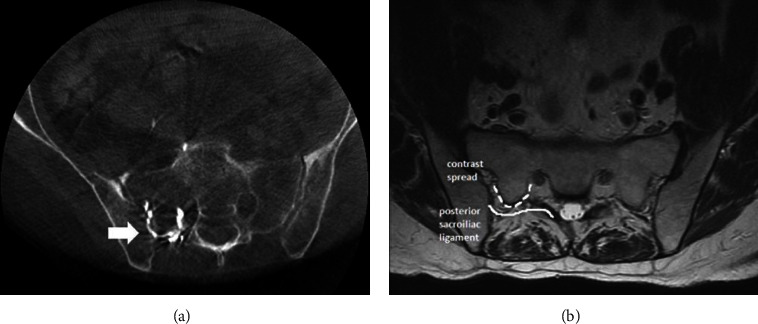
3D-CBCT images (a)—Arrow indicating contrast spread in the right sacroiliac joint tracking into the right posterior S1 neuroforamen. The similar level axial plane on the lumbar spine MRI (b) demonstrating posterior sacroiliac ligament (solid line) which corresponds to this contrast track (dotted line indicating the track through which contrast spreads from the sacroiliac joint into posterior S1 neuroforamen).

Thirty-two patients with sacroiliac joint dysfunction underwent SI joint injections during the study. Five patients were excluded from this study based on exclusion criteria (Figure1).Twenty-seven patients, mean age, 56 years old (range 39–87 years) old, twenty females (74.1%), and seven males (25.9%), were included in the study. After reviewing the postinjection cone beam CT images, we observed that 4/27 (14.8%) patients had contrast spread to posterior S1 neuroforamen. The remaining 23/27 (85.2%) patients showed localized contrast spread within the sacroiliac joint. The images of three of the four patients with contrast spread in posterior S1 neuroforamen are shown in Figures 2, 3, 4.

In Figure 2, patients A and B: 3D-CBCT showed contrast in the sacroiliac joints with spread into the posterior S1 neuroforamen.  Figure 3, patient C: fluoroscopy image showed contrast from sacroiliac joint spread to left posterior S1 neuroforamen. 3D-CBCT images in the axial and coronal views demonstrate contrast spread to the posterior S1 neuroforamen. In Figure 4, patient D: 3D-CBCT showed contrast in the sacroiliac joints with spread into the posterior S1 neuroforamen. We were able to find this patient's prior lumbar MRI and compared similar level axial plane of MRI to post-SI injection CT. In the MRI image, the dotted line indicates the track underneath the posterior sacroiliac ligament through which contrast spreads from the sacroiliac joint into posterior S1 neuroforamen.

## 4. Discussion

The sacroiliac joint is a diarthrodial joint secured by intrinsic and extrinsic ligaments. The intrinsic ligaments of the joint, including the anterior sacroiliac ligament, the posterior sacroiliac ligament, and the interosseous ligament connect the iliac to the sacrum [18]. The posterior sacroiliac ligament runs from the posterior superior iliac spine (PSIS) to the various posterior segments of the sacrum. Running superiorly from the iliac tuberosity to the sacrum is the interosseous ligament. The largest recess is the upper portion of the sacroiliac joint, while the smallest recess is the lowest portion of the SI joint. In this case series, a CT scan was conducted to visualize joint space, and we use this CT image to select a relatively large opening of the joint for injection. According to Dreyfuss's study, 75% of its superior joint surface is not synovial [19]. We observed that the largest recess is in the superior one third of sacroiliac joint where interosseous ligaments and posterior sacroiliac ligaments are located. Below this recess, in the middle portion of the joint allows easier access for synovial part of the joint space. In order to position the needles in the synovial part of the joint space, we selected our target at the junction of superior one-third and inferior two-thirds of the joint space (see Figure 3), which is an area with a relatively large posterior joint opening, and in the synovial part of the joint space. This approach has previously been described in the Spine Intervention Society Practice Guidelines in 2014 and later published by Kyung Hee Do et al. [20, 21]. This is different from the inferior approach that is commonly adopted by most pain specialists.

In this case review, we observed that the contrast from SI joint to S1 posterior neuroforamen. We compared magnetic resonance imaging (MRI) and CT scans and discovered a track underneath posterior sacroiliac ligament from the ilium to the sacrum through which contrast traveled from SI joint to S1 posterior neuroforamen. In Figure 4, cone beam CT demonstrates contrast spread in the left sacroiliac joint tracking into the left posterior S1 neuroforamen. On the same axial plane, lumbar spine MRI demonstrates posterior sacroiliac ligament which corresponds to this contrast track. Our findings through this review provided clear evidence that injectates can spread underneath the posterior sacroiliac ligament and reach the posterior S1 neuroforamen and into the epidural space. Conceivably, the local anesthetic injected can inadvertently produce an S1 nerve root block.

In this case series, we observed that approximately 15% patients had contrast spread to S1 neuroforamen. It is worth noting that one milliliter of contrast was injected in the SI joint before CT scan. If more contrast was injected, we might observe more contrast spread in S1 posterior neuroforamen and more cephalad spread in the epidural space. In addition, after an injection of a mixture containing 3 ml of 0.25% bupivacaine and 1 ml betamethasone (6 mg), none of the patients reported weakness in the lower extremities. However, this does not mean there was no sensory block of S1 nerve root from the injections because no sensory examination was conducted after the injections. The patients were discharged home per PACU discharge criteria, which does not include sensory examination. We suspect some of these patients had some degree of sensory block that were not documented since no sensory data were collected.

Surgical stabilization and fusion of the sacroiliac joint are often considered when a patient has failed conservative treatments with continued severe pain and functional impairments. There is mounting evidence that SI joint fusion can provide significant pain, disability, function, and quality of life outcomes compared to conservative treatments [22–24]. A positive SI joint injection is one of the important diagnostic criteria for SI fusion. Our findings indicate that there is potential local anesthetic spread to S1 neuroforamen and epidural space from diagnostic SI injections, which may lead to a false positive result for patients with pain from S1 pathology and lumbar spine, especially lower segment of the lumbar spine. To minimize the false positive rate, an appropriate evaluation and diagnostic tests should be considered to rule out pain from S1 nerve root and lower segment of lumbar spine prior to diagnostic SI joint injections.

There are limitations to this study. The major limitation is the study design, which is a retrospective review of medical records. The second limitation is that no sensory and motor examinations of lower extremities were documented in the PACU records after SI joint injections. The patients were discharged home after injections if they reported no lower extremity weakness and were able to walk per PACU discharge criteria. Another limitation is the small number of cases in this series.

## 5. Conclusion

This study demonstrated that injected contrast could track from SI joint, underneath posterior sacroiliac ligament, into the posterior S1 neuroforamen, which may lead to S1 nerve root block and possible epidural block. However, this is a retrospective case review study with a small sample size, and none of the patients in this study reported symptoms of S1 sensory and motor block. Future research with larger-scale, well-designed prospective studies are required to determine its clinical relevance. Based upon current results, we speculate that the injection of lower concentration of local anesthetic with less volume may be necessary to decrease the potential risk of S1 nerve root block and epidural block. In addition, to improve the specificity of a diagnostic SI injection, an appropriate evaluation should be considered to rule out any S1 nerve pathology as a significant pain generator.

## Data Availability

The data that support the findings of this study are available from the corresponding author upon reasonable request.
